# Silica-supported sulfonic acids as recyclable catalyst for esterification of levulinic acid with stoichiometric amounts of alcohols

**DOI:** 10.3762/bjoc.12.207

**Published:** 2016-10-12

**Authors:** Raimondo Maggi, N Raveendran Shiju, Veronica Santacroce, Giovanni Maestri, Franca Bigi, Gadi Rothenberg

**Affiliations:** 1 Clean Synthetic Methodology Group, Dipartimento di Chimica, Università di Parma, Parco Area delle Scienze 17A, I-43124 Parma, Italy; 2 Van ’t Hoff Institute for Molecular Sciences, University of Amsterdam, Science Park 904, 1098 XH, Amsterdam, The Netherlands. Tel: +31-20-5256515; 3 Istituto IMEM-CNR, Parco Area delle Scienze 37/A, I-43124 Parma, Italy

**Keywords:** esterification, heterogeneous catalysis, renewable feedstocks, supported organic catalysts, sustainable chemistry

## Abstract

Converting biomass into value-added chemicals holds the key to sustainable long-term carbon resource management. In this context, levulinic acid, which is easily obtained from cellulose, is valuable since it can be transformed into a variety of industrially relevant fine chemicals. Here we present a simple protocol for the selective esterification of levulinic acid using solid acid catalysts. Silica supported sulfonic acid catalysts operate under mild conditions and give good conversion and selectivity with stoichiometric amounts of alcohols. The sulfonic acid groups are tethered to the support using organic tethers. These tethers may help in preventing the deactivation of the active sites in the presence of water.

## Introduction

Vegetal biomass is mankind’s only source of renewable carbon on a human timescale. It is abundantly available, with the potential of replacing fossil-based carbon on a scale sufficient for covering the worldwide demand for non-fuel chemicals [1–4]. Currently, the main research thrust is directed at lignocellulose, the most abundant fraction of biomass. The mass composition of lignocellulose could be roughly represented by a 5/3/2 ratio of cellulose, hemicellulose and lignin, respectively. All of these polymers are the subject of many studies [5–11].

**Scheme 1:**
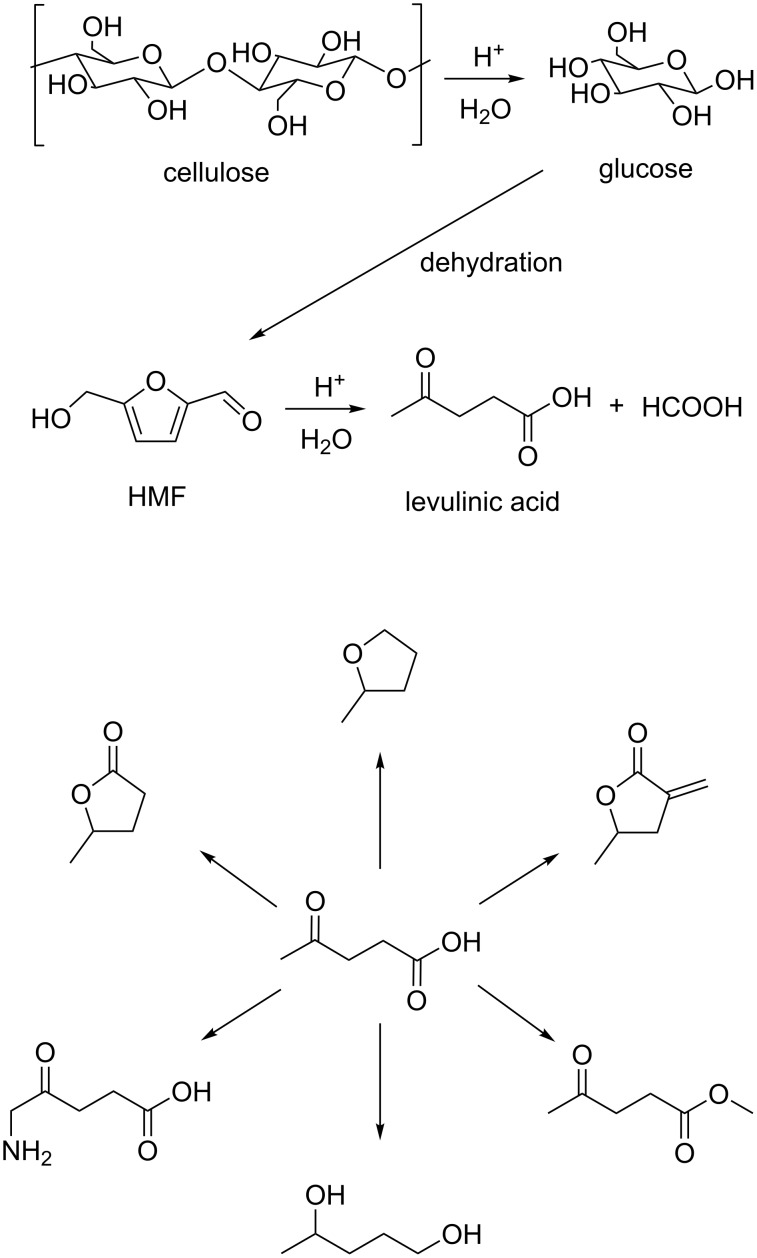
Synthesis of levulinic acid from ligno-cellulosic feedstocks and its principal uses to access fine chemicals.

Levulinic acid (LA) is one of the most important platform chemicals as it is a versatile building block for a variety of value-added agrochemicals, fine chemicals and pharmaceutical intermediates [12–13] (Scheme 1, bottom). Moreover, it can be obtained from cellulose with relative ease and high selectivity (see Scheme 1, top) [14].

Levulinic acid esters are of particular interest for the chemical industry [12–13]. Their main current market is represented by the formulation of flavours and fragrances [15], although the scale of these preparations did not boosted demand yet. However, the seek to develop more eco-compatible solvents might grant to levulinates a novel route of application. By tailoring their physicochemical properties they could become complementary to common esters and other solvents, which might be more harmful for both humans and the environment [16]. It should be also noted that ethyl levulinate could shrink the emission of nitrogen oxides from exhausts of diesel engines when used as additive [17–18].

Due to their importance, new strategies have been developed for the production of levulinic esters [19–22]. Homogeneous Brønsted acids could catalyse the esterification of levulinic acid in the presence of alcohols and reports on this reactivity date back to the nineties [23]. Although this route could ensure high chemical yields, it still presents a series of drawbacks. In particular, issues with catalyst recycling and product separation limits the environmental viability of this strategy. As a result, it remains of high interest to develop alternatives to trigger this reaction, which are more sustainable, for instance through the design of suitable and recyclable solid acid catalysts. In the literature, methods that use solid heteropolyacids, such as ammonium or mixed ammonium and silver-doped phosphotungstic acid, sulfated metal oxides (such as sulfated titania, sulfated zirconia), zeolites and hydrotalcites have been reported [24–30]. These solid catalysts share several advantages, including high activity and an easy recovery, which might provide a real basis for future application in commercial processes. Nevertheless, they require high temperatures (usually above 100 °C) and long reaction times [24–30]. Furthermore, they often share another common pitfall, namely the use of large molar excess of alcohol, either for practical convenience [31] or to minimise ester hydrolysis. As meaningful examples, it has been recently reported that acid ZSM-5 zeolites, with encapsulated maghemite particles to allow magnetic catalyst recover, could be used to directly convert furfuryl alchol into an alkyl levulinate upon warming at 130 °C for 8 hours in the presence of a large excess of alchol as solvent/reagent (100 equiv) [32]. Although the behaviour of many metal oxides has been investigated, reports featuring the activity of supported organic Brønsted acids are very few. In particular, Tejero reported that sulfonic acid supported on polymeric resins could catalyse the esterification of LA, providing conversions up to 94% upon warming at 80 °C for 8 hours in the presence of 3 equiv of *n*-butanol [33]. Melero described the synthesis of mesostructured silica frameworks featuring pending organosulfonic arms. The best catalyst provided quantitative conversion of LA upon warming of the reaction mixture at 130 °C for 2 hours in the presence of a five-fold molar excess of ethanol, used as solvent/reagent [34].

Here we present an alternative strategy in which a heterogeneous catalyst triggers the selective esterification of levulinic acid with a stoichiometric amount of alcohol.

**Figure 1:**
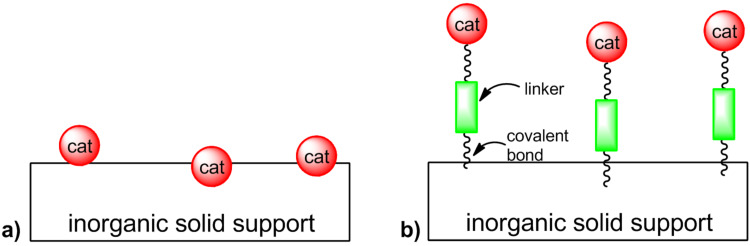
Anchoring methodologies: a) impregnation; b) covalent binding.

In the last years, many methods have been developed for the transformation of homogeneous catalysts into recyclable heterogeneous ones. To prevent leaching, a common strategy is tethering the active species with the support via covalent bonds [35]. This approach increases the stability of the catalyst itself compared to impregnation (Figure 1). Furthermore, the activity of the catalyst can be tuned through adoption of a suitable linker.

## Results and Discussion

As part of our interest in acid catalysis [36–38], we prepared a set of solid materials for the esterification of levulinic acid. Upon preliminary screening, supported sulfonic acids seemed promising candidates. They were prepared following a reported procedure by the tethering method [39], which consists of the immobilisation of a functional moiety on an inorganic support via covalent bonds ensured by a suitable linker [35].

In preliminary experiments reactions were carried out with a five-fold molar excess of alcohol. We started from this ratio as in the literature we did not retrieve any catalytic method for the esterification of biomass-derived acids that operates with a lower molar excess of alcohol [24–34]. 1-Pentanol was selected as model substrate in order to work over an ample range of operating temperatures. Thus, in a typical experiment, 10 mmol of levulinic acid were stirred at 100 °C in a sealed tube for 2 h under air in the presence of the amount of a solid catalyst necessary to have 1 mol % of acid sites. The results are reported in Table 1.

**Table 1:** Screening of different solid acids in the esterification reaction of levulinic acid with 1-pentanol.

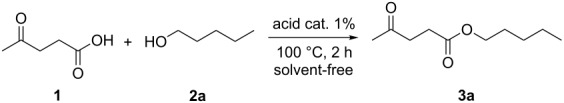					

Entry	Sulfonated catalyst	Catalyst acidity (mmol H^+^/g)	Conversion of **1** (%)	Yield of **3a** (%)	Selectivity of **3a** (%)

1	SiO_2_-(CH_2_)_3_-O-C_6_H_4_-SO_3_H	0.73	94	84	89
2	SiO_2_-C_6_H_4_-SO_3_H	0.65	92	90	98
3	SiO_2_-(CH_2_)_3_-SO_3_H	0.51	95	93	98
4^a^	SiO_2_-(CH_2_)_3_-SO_3_H	0.51	96	94	98
5	Amberlyst 15	4.70	52	31	60
6	Nafion^®^	0.80	72	68	94
7	Aquivion^®^	0.12	84	80	95
8	H_2_SO_4_		57	55	96

^a^With the addition of 4 Å molecular sieves. Values by GC upon calculation of response factors for **1** and **3a** from pure samples over the concentration interval of the reaction; the selectivity has been calculated as the ration between yield of **3a** and conversion of **1**.

All of the prepared silica-supported sulfonic acids showed very good catalytic activity for the esterification of levulinic acid (Table 1, entries 1–4). Materials with an arylsulfonic moiety were initially investigated (Table 1, entries 1 and 2). They present a comparable loading of Brønsted sites (0.73 and 0.65 mmol/g respectively) and ensured conversion of **1** above 90% within two hours (94 and 92%). The catalyst without any alkyl tether was more selective, ultimately delivering the desired product **3a** in 90% yield. Silica-supported propyl sulfonic acid provided slightly better results (Table 1, entries 3 and 4). The material presented a lower density of Brønsted sites (0.51 mmol/g), but delivered almost complete conversion of **1** within 2 h (>95%), affording **3a** in 93% yield. We then repeated the experiment adding activated molecular sieve in the reaction flask (Table 1, entry 4) to check whether water coproduced by the reaction could cause any harm. The outcome paralleled the standard procedure, conversion and yield being 96% and 94%, respectively. This result shows that the presence of water is tolerated by the catalytic system, which in turn did not easily trigger the hydrolysis of levulinates under these conditions.

Remarkably, the presence of water on the catalyst surface can inhibit the catalytic sites of inorganic materials instead [40]. Acidic and/or hydrophilic metal oxides and sulfates easily adsorb water on their surface, which is the coproduct of the esterification. This can severely reduce the activity of the catalyst. Considering our supported sulfonic acids, we speculate that their organic tethers could smooth the hydrophilic character of their Brønsted sites and thus prevent the deactivation due to water.

We then tested a selection of commercial catalysts (Table 1, entries 5–7). Despite encouraging literature precedents [22,33], Amberlyst 15 gave only 52% conversion of **1** within 2 h (Table 1, entry 5, 31% yield). We then switched to perfluorinated resins. Nafion^®^ and Aquivion^®^ showed an interesting selectivity towards **3a**, but conversion of **1** proved once again below that observed with supported sulfonic acids (72% and 84%, respectively). Finally, a common homogeneous acid was used for comparison. The use of 1 mol % of H_2_SO_4_ (Table 1, entry 8) delivered **3a** in 55% yield only. Furthermore, conversion of **1** remained stuck at 57% even prolonging the reaction time for up to 24 h. This result shows that heterogeneous sulfonic acids outperform their homogeneous peer under these conditions.

Upon identification of silica-supported sulfonic acids as cheap and promising candidates for the selective esterification of LA, the reaction parameters were then optimized in order to maximise the environmental viability of the method. We thus tried to shelve the molar excess of the alcohol (Table 2).

**Table 2:** Variation of the acid/alcohol ratio.

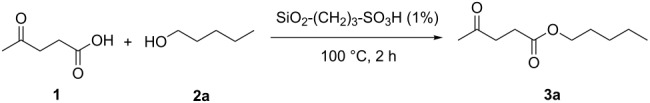				

Entry	Acid:alcohol ratio	Conversion of **1** (%)	Yield of **3a** (%)	Selectivity of **3a** (%)

1	1:5	96	94	98
2	1:2	95	93	98
3	1:1	96	94	98

Reactions were carried out at 100 °C and regularly monitored for 2 h. To our delight, varying the amount of alcohol did not hamper neither conversion nor selectivity. Indeed, **3a** was recovered in 93% yield using a two-fold molar excess of **2** (Table 2, entry 2). A comparable result was achieved with a stoichiometric amount of pentanol (Table 2, entry 3, 94% yield). It is remarkable that even in this case the amount of water coproduced by the esterification did not cause any significant hydrolysis of the desired ester **3a**. Furthermore, the almost complete conversion of **1** with a stoichiometric amount of **2a** allows minimising the consumption of reagents and therefore the overall costs of the transformation. To the best of our knowledge, using stoichiometric amounts of alcohol has not been reported previously. In the present case, this can be possible as we could show that a relatively high concentration of water did not hinder the reaction. Catalysts more prone to deactivation might require a larger molar excess of alcohols to prevent water-poisoning.

The reaction conditions were further optimized studying the effect of the temperature. So, a series of experiments were carried out using the sulfonated catalyst (1%), an equimolecular amount of reagents under solvent free conditions, and varying the temperature between 50–100 °C. Results are reported in Table 3.

**Table 3:** Variation of the reaction temperature.

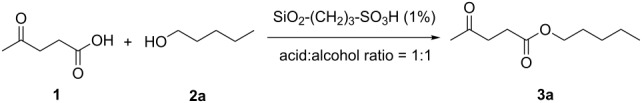					

Entry	Temperature (°C)	Conversion of **1** (%)	Yield of 3**a** (%)	Selectivity of **3a** (%)	Time (h)

1	100	96	94	98	2
2	75	95	93	98	2
3	50	79	77	97	7

In all cases, the selectivity towards the esterification product **3a** remained complete. A comparable yield of **3a** was recovered reducing the temperature from 100 to 75 °C (Table 3, entry 2, 93%). By reducing the temperature to 50 °C (Table 3, entry 3), longer reaction times became necessary. At 50 °C, conversion peaked at 79% upon 7 h and no longer improved even by keeping the mixture for further 24 h. Reasoning on the practical viability of the method, we therefore continued our study fixing the temperature at 75 °C. We then evaluated the amount of catalyst (Table 4).

**Table 4:** Effect of the amount of catalyst.

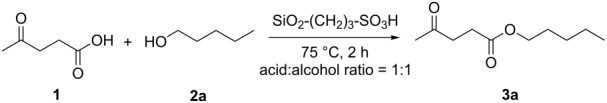				

Entry	Catalyst amount (%)	Conversion of **1** (%)	Yield of **3a** (%)	Selectivity of **3a** (%)

1	5	95	85	89
2	1	95	93	98
3	0.1	36	35	97
4	0.01	15	14	93

Surprisingly, an increase of the catalyst amount to 5 mol % resulted in lower selectivity towards **3a**, which has been retrieved in 85% yield together with traces of one unidentified byproduct (Table 4, entry 1). On the other hand, reduction of the catalyst loading to 0.1 mol % slows down the process, conversion being just 36% upon 2 h (Table 4, entry 3). Further reduction to 0.01 mol % confirmed this trend and delivered **3a** in 14% yield (Table 4, entry 4). Even by prolonging the reaction time to 24 h, conversion did not reach completion and the ester was isolated in 58 and 38% yield with 0.1 and 0.01 mol % of catalyst, respectively. In any case, the selectivity remained almost complete (>98% by GC).

**Figure 2:**
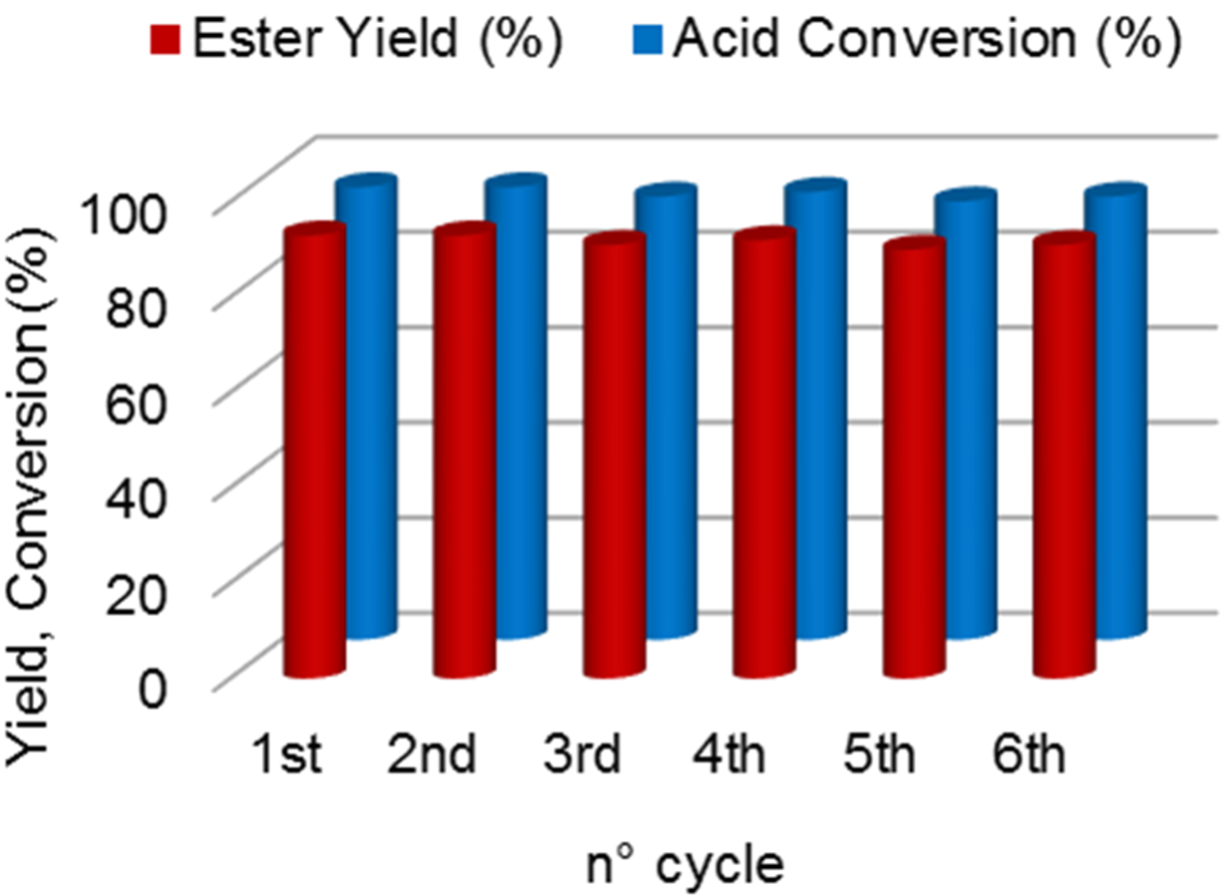
Activity of the supported sulfonic acid catalyst within the first six cycles. Reaction conditions: 1 mol % cat., acid:alcohol ratio = 1:1, solvent free, 75 °C, 2 h.

We then ensured that the catalyst acts as a heterogeneous species by performing a filtration test. In agreement with the hypothesis, we monitored no further conversion on the filtrate [41], proving that no leaching occurred. The recyclability of the catalyst was then evaluated. The catalyst was recovered by filtration, washed with ethyl acetate (10 mL), dried and reused for a further esterification. The results are shown in Figure 2.

The catalyst can be recovered and reused for 6 cycles at least, fully preserving its activity and selectivity. For instance, conversion of **1** and yield of **3a** were 94 and 92%, respectively, upon the fifth re-cycle.

Finally, with optimized conditions in our hands, we checked the scope of this catalytic methodology (Table 5).

**Table 5:** Esterification of levulinic acid with different alcohols.

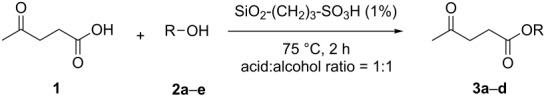				

Entry	Alcohol	Conversion of **1** (%)	Yield of **3** (%)^a^	Selectivity of **3** (%)

1	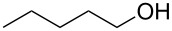 **2a**	95	93	98
2^b^	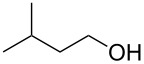 **2b**	80	79	99
3	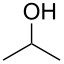 **2c**	60	59	98
4	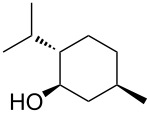 **2d**	77	76	99
5^c^	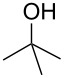 **2e**	0	0	–

^a^Isolated yields upon chromatography; ^b^by warming for 5 h; ^c^by warming for 24 h.

As expected, the best performances were obtained with primary alcohols (Table 5, entries 1 and 2), which afforded the desired ester in 93 and 79% yield, respectively. Gratifyingly, the method could be extended to secondary alcohols as isopropanol and L-menthol. Despite their increased steric hindrance, very good results were obtained with a selectivity towards **3** >99% (Table 5; entries 3 and 4, 59 and 76% yield). In particular, it is important to underline that a single diastereomer of product **3d** was formed (Table 4, entry 4). This implies that the present method, likely thanks to its mild conditions, allows to preserve chiral information present on substrates and could thus efficiently transfer it on the products.

On the other hand, no conversion of **1** was observed using tertiary alcohols, as witnessed by entry 5. Probably, their steric hindrance quenches any reactivity.

## Conclusion

Silica-supported sulfonic acids proved very active heterogeneous catalysts for the selective esterification of levulinic acid with stoichiometric amounts of primary alcohols. The esterification can be carried out under mild conditions (75 °C, 2 h) and provides good to excellent yields with various primary and secondary alcohols. The selectivity towards desired products remained complete in all cases. The coproduct of the reaction, namely water, did not hamper the efficiency of this solvent-free process.

The selected catalyst is cheap, can be easily prepared from commercial reagents and proved very robust. It is very active and selective, water-tolerant and recyclable. It represents therefore an interesting and complementary alternative to existing esterification catalysts. Together with the absence of solvents and of any molar excess of reagents, these features highlight the practical and environmental viability of this catalytic method.

## Experimental

### Catalysts preparation

**SiO_2_-(CH_2_)_3_-SO_3_H** [35]: Amorphous silica (8.0 g) has been refluxed under stirring for 24 h with (3-mercaptopropyl)trimethoxysilane (MPTS) (1.15 mL; 6.1 mmol) in toluene (120 mL) and the resulting supported propylmercaptane has been oxidized to propanesulfonic acid by treatment with 30% aq H_2_O_2_ (100 mL; 1 mol) for 24 h under stirring at rt, adding a few drops of concentrated sulfuric acid after 12 h. Acidity has been measured via the titration method [35] (0.51 mmol H^+^/g).

**SiO_2_-C_6_H_4_-SO_3_H** [35]: Amorphous silica (8.0 g) has been refluxed in toluene (120 mL) with phenyltriethoxysilane (2.0 mL, 8.3 mmol) under stirring for 24 hours. The resulting solid was then filtered off and washed with toluene (3 × 20 mL). The supported phenyl group was then sulfonated by refluxing in 1,2-dichloroethane (60 mL) the functionalized material with cholorosulfonic acid (10 mL, 150 mmol) under stirring for 4 hours. The solid was then recovered by filtration and washed with 1,2-dichloroethane (3 × 20 mL), acetone (3 × 20 mL) and water (3 × 50 mL) to deliver the title compound. Acidity has been measured via the titration method [35] (0.65 mmol H^+^/g).

**SiO_2_-(CH_2_)_3_-O-C_6_H_4_-SO_3_H:** A mixture of amorphous silica gel (2.0 g) and bromopropyltrimethoxysilane (0.76 mL, 4.0 mmol) was refluxed in toluene (80 mL) under stirring for 24 hours. The resulting silica supported 3-bromopropane was recovered by filtration and washed with toluene (3 × 50 mL). A mixture of this material (2.0 g) and sodium phenoxide (0.6 g, 6.0 mmol) in DMF (100 mL) was then heated at 100 °C under stirring for 24 hours. Afterwards, the material was filtered, washed with DMF (3 × 20 mL) and acetone (3 × 20 mL). The resulting solid material (2.0 g) and chlorosulfonic acid (4 mL, 60 mmol) were eventually stirred in refluxing 1,2-dichloroethane (60 mL) under stirring for 4 hours. The catalyst was then recovered by filtration and washed with 1,2-dichloroethane (3 × 20 mL), acetone (3 × 20 mL) and water (3 × 50 mL). Acidity has been measured via titration method [35] (0.73 mmol H^+^/g).

### Esterification reaction

Levulinic acid, pentanol and the heterogeneous catalyst were stirred for 24 hours in a batch reactor under air. The acid/alcohol ratio, the reaction temperature and the amount of the catalyst were modified as described in the previous section. In all cases, the solid catalyst was eventually recovered by filtration and the reaction mixture was analysed by high resolution capillary GC with a fused silica capillary column SE52 (5% phenyl, 95% methyl polysiloxane, 30 m × 25 mm). The products were isolated by flash chromatography on silica gel (eluent = hexane/ethyl acetate) and characterised by multinuclear NMR.

## Supporting Information

File 1 Experimental part and NMR spectra of products.
