# Supplementary material for: Silica-supported sulfonic acids as recyclable catalyst for esterification of levulinic acid with stoichiometric amounts of alcohols
Source: Beilstein J Org Chem. 2016 Oct 12;12:2173–80. doi: 10.3762/bjoc.12.207 (PMC5082645; doi:10.3762/bjoc.12.207)
Supplement: File 1 — Experimental part and NMR spectra of products. [file Beilstein_J_Org_Chem-12-2173-s001.pdf]

Supporting information

for

Silica-supported sulfonic acids as recyclable catalyst for esterification of levulinic acid with stoichiometric amounts of alcohols

Raimondo Maggi*[1], N. Raveendran Shiju*[2], Veronica Santacroce[1,2], Giovanni Maestri[1], Franca Bigi[1,3] and Gadi Rothenberg[2]

Address: [1]Clean Synthetic Methodology Group, Dipartimento di Chimica, Università di Parma, Parco Area delle Scienze 17A, I-43124 Parma, Italy, [2]Van ’t Hoff Institute for Molecular Sciences, University of Amsterdam, Science Park 904, 1098 XH, Amsterdam, The Netherlands. Tel: +31-20-5256515 and [3]Istituto IMEM-CNR, Parco Area delle Scienze 37/A, I-43124 Parma.

Email: Raimondo Maggi - raimondo.maggi@unipr.it; N. Raveendran Shiju - n.r.shiju@uva.nl

*Corresponding author

Experimental part and NMR spectra of products

Contents:

S3: General remarks

S4: Catalysts preparation

S6: Catalytic esterification of levulinic acid

S7: Characterization of products

S9: Copies of ^{1}H and ^{13}C NMR spectra

General remarks

Reagents were obtained from commercial sources and used as received.

Flash column chromatography was performed on Merck Geduran silica gel 60 (0.040−0.063 mm) and thin-layer chromatography on Merck 60F 254 plates.

^{1}H NMR and ^{13}C NMR NMR spectra were recorded in $CDCl_3$ at 298 K on a Bruker 400 AVANCE spectrometer using the solvent as internal standard. The terms m, s, d, t, q represent multiplet, singlet, doublet, triplet, quadruplet, respectively. All products are known compounds and their spectroscopic data correspond to the literature.

Exact masses of products 3a-3d were recorded on a LTQ ORBITRAP XL Thermo Mass Spectrometer (electrospray source).

Catalysts preparation

- SiO_2-$(CH_2)_3$-SO_3H: amorphous silica (8.0 g) has been refluxed in toluene (120 mL) with (3-mercaptopropyl)trimethoxysilane (MPTS) (1.15 mL, 6.1 mmol) under stirring for 24 hours. The mixture was then filtered and the resulting solid, namely supported propylmercaptane has been oxidized to propanesulfonic acid by treatment with 30% aq H_2O_2 (100 mL, 1 mol) under stirring for 24 hours at rt, adding a few drops of concentrated sulfuric acid after 12 hours. The white solid was then filtered and dried in vacuum. Its acidity has been measured by titration with an aqueous NaOH solution.

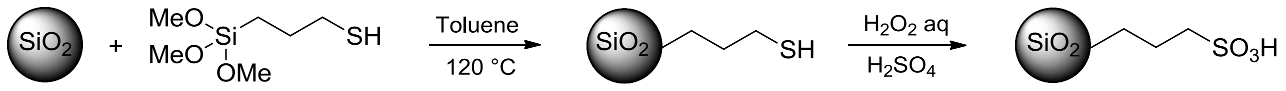

- SiO_2-C_6H_4-SO_3H: amorphous silica (8.0 g) has been refluxed in toluene (120 mL)with phenyltriethoxysilane (2.0 mL, 8.3 mmol) under stirring for 24 hours. The resulting solid was then filtered off and washed with toluene. The supported phenyl group was then sulfonated by refluxing in 1,2-dichlotoethane (60 mL) the material with cholorosulfonic acid (10 mL, 150 mmol) under stirring for 4 hours. The solid was then recovered by filtration and washed carefully with 1,2-dichloroethene (3 × 20 mL), acetone (3 × 20 mL) and water (3 × 50 mL).

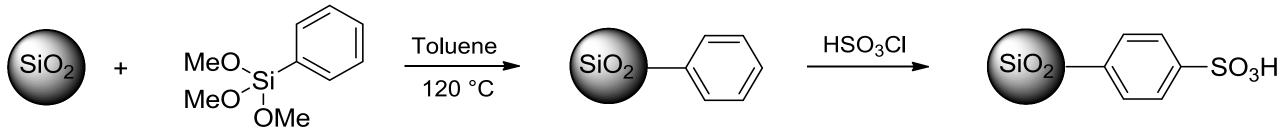

- SiO_2-$(CH_2)_3$-O-C_6H_4-SO_3H: a mixture of amorphous silica gel (2.0 g) and bromopropyltrimethoxysilane (0.76 mL, 4.0 mmol) was refluxed in toluene (80 mL) under stirring for 24 hours. The resulting silica supported 3-bromopropane was recovered by filtration and washed with toluene (3 × 50 mL). A mixture of this material (2.0 g) and sodium phenoxide (0.6 g, 6.0 mmol) in DMF (100 mL) was heated to 100 °C under stirring for 24 hours. Afterwards the material was filtered and washed with DMF (3 × 20 mL) and acetone (3 × 20 mL). The resulting solid (2.0 g) and chlorosulfonic acid (4 mL, 60 mmol) were stirred in refluxing 1,2-dichloroethane (60 mL) for 4 hours. The catalyst was then recovered by filtration and washed with 1,2-diclhorothene (3 × 20 mL), acetone (3 × 20 mL) and water (3 × 50 mL).

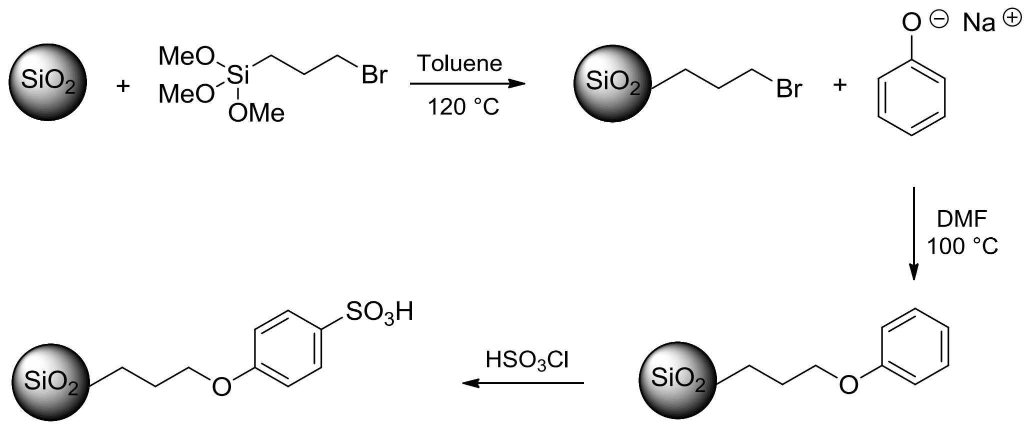

Catalytic levulinic acid esterification

In a stirred batch reactor levulinic acid, pentanol and the heterogeneous catalyst were stirred for 24 hours. The acid/alcohol ratio, as well as the reaction temperature and the amount of the catalyst were changed as described in Tables 2, 3 and 4. Then the solid catalyst was recovered by filtration and the reaction mixture was analysed by high-resolution capillary GC equipped with a fused silica capillary column SE52 (5% phenyl, 95% methyl polysiloxane, 30 m × 25 mm). The identity and purity of products has been further checked by NMR.

Characterization of products

(**3a**) Pentyl 4-oxopentanoate

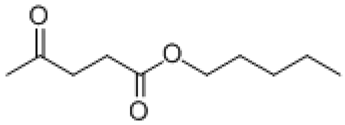

$C_{10}H_{18}O_3$ MW = 186.25 g/mol

According to the general procedure described above, **3a** has been synthesized from levulinic acid (1.0 mL, 10 mmol) and pentanol (1.1 mL, 10 mmol). Isolated as a colorless oil. Yield: 93%.

^{1}H NMR (400 MHz, $CDCl_3$) δ = 4.02 (t, J = 6.7 Hz, 2H), 2.71 (t, J = 6.5 Hz, 2H), 2.53 (t, J = 6.5 Hz, 2H), 2.15 (s, 3H), 1.60-1.56 (m, 2H), 1.30-1.28 (m, 4H), 0.88-0.85 (m, 3H).

^{13}C NMR (100.5 MHz, $CDCl_3$) δ = 206.5, 172.7, 64.7, 37.9, 29.7, 28.2, 27.92, 27.90, 22.2, 13.8.

HRMS calculated for $C_{10}H_{19}O_3^+$ [M+H$^+$]: 186.1256, found: 186.1252.

(**3b**) Isopentyl 4-oxopentanoate

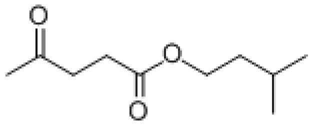

$C_{10}H_{18}O_3$ MW = 186.25 g/mol

According to the general procedure described above, **3b** has been synthesized from levulinic acid (1.0 mL, 10 mmol) and isoamyl alcohol (1.1 mL, 10 mmol). Isolated as a colorless oil. Yield: 79%.

^{1}H NMR (400 MHz, $CDCl_3$) δ = 4.08 (t, J = 6.8 Hz, 2H), 2.73 (t, J = 6.4 Hz, 2H), 2.55 (t, J = 6.5 Hz, 2H), 2.17 (s, 3H), 1.70-1.60 (m, 1H), 1.52-1.47 (m, 2H), 0.91-0.89 (m, 6H).

^{13}C NMR (100.5 MHz, $CDCl_3$) δ = 206.7, 172.8, 63.3, 37.9, 37.2, 29.8, 28.0, 25.0, 22.4.

HRMS calculated for $C_{10}H_{19}O_3^+$ [M+H$^+$]: 186.1256, found: 186.1254.

(**3c**) Isopropyl 4-oxopentanoate

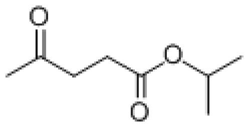

$C_8H_{14}O_3$ MW = 158.19 g/mol

According to the general procedure described above, **3c** has been synthesized from levulinic acid (1.0 mL, 10 mmol) and isopropyl alcohol (0.8 mL, 10 mmol). Isolated as a colorless oil. Yield: 59%.

^{1}H NMR (400 MHz, $CDCl_3$) δ = 4.99 (hept, J = 6.2 Hz, 1H), 2.73 (t, J = 6.6 Hz, 2H), 2.54 (t, J = 6.6 Hz, 2H), 2.19 (s, 3H), 1.23 (d, J = 6.3 Hz, 6H).

^{13}C NMR (100.5 MHz, $CDCl_3$) δ = 206.8, 172.2, 68.0, 38.0, 29.9, 28.4, 21.8.

HRMS calculated for $C_8H_{15}O_3^+$ [M+H$^+$]: 158.0943, found: 158.0939.

(**3d**) (1R,2S,5R)-2-Isopropyl-5-methylcyclohexyl 4-oxopentanoate

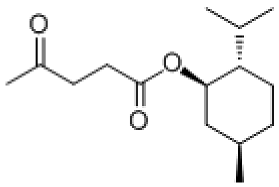

$C_{15}H_{26}O_3$ MW = 254.37 g/mol

According to the general procedure described above, **3d** has been synthesized from levulinic acid (1.0 mL, 10 mmol) and menthol (1.6 g, 10 mmol). Isolated as a colorless oil. Yield: 76%.

^{1}H NMR (400 MHz, $CDCl_3$) δ = 4.64 (td, J = 10.9, 4.4 Hz, 1H), 2.71 (t, J = 6.6 Hz, 2H), 2.52 (t, J = 6.5 Hz, 2H), 2.15 (s, 3H), 1.97 – 1.91 (m, 1H), 1.83 (ddd, J = 13.9, 7.0, 2.7 Hz, 1H), 1.65-1.55 (m, 3H), 1.48-1.38 (m, 1H), 1.37-1.30 (m, 1H), 1.11-0.95 (m, 2H), 0.87 (dd, J = 6.4, 1.3 Hz, 6H), 0.72 (d, J = 7.0 Hz, 3H).

^{13}C NMR (100.5 MHz, $CDCl_3$) δ = 206.6, 172.2, 74.4, 46.9, 40.8, 38.0, 34.2, 31.3, 28.7, 26.2, 23.4, 21.9, 20.6, 16.3.

HRMS calculated for $C_{15}H_{27}O_3^+$ [M+H$^+$]: 254.1882, found: 254.1876.

^{1}H and ^{13}C NMR spectra

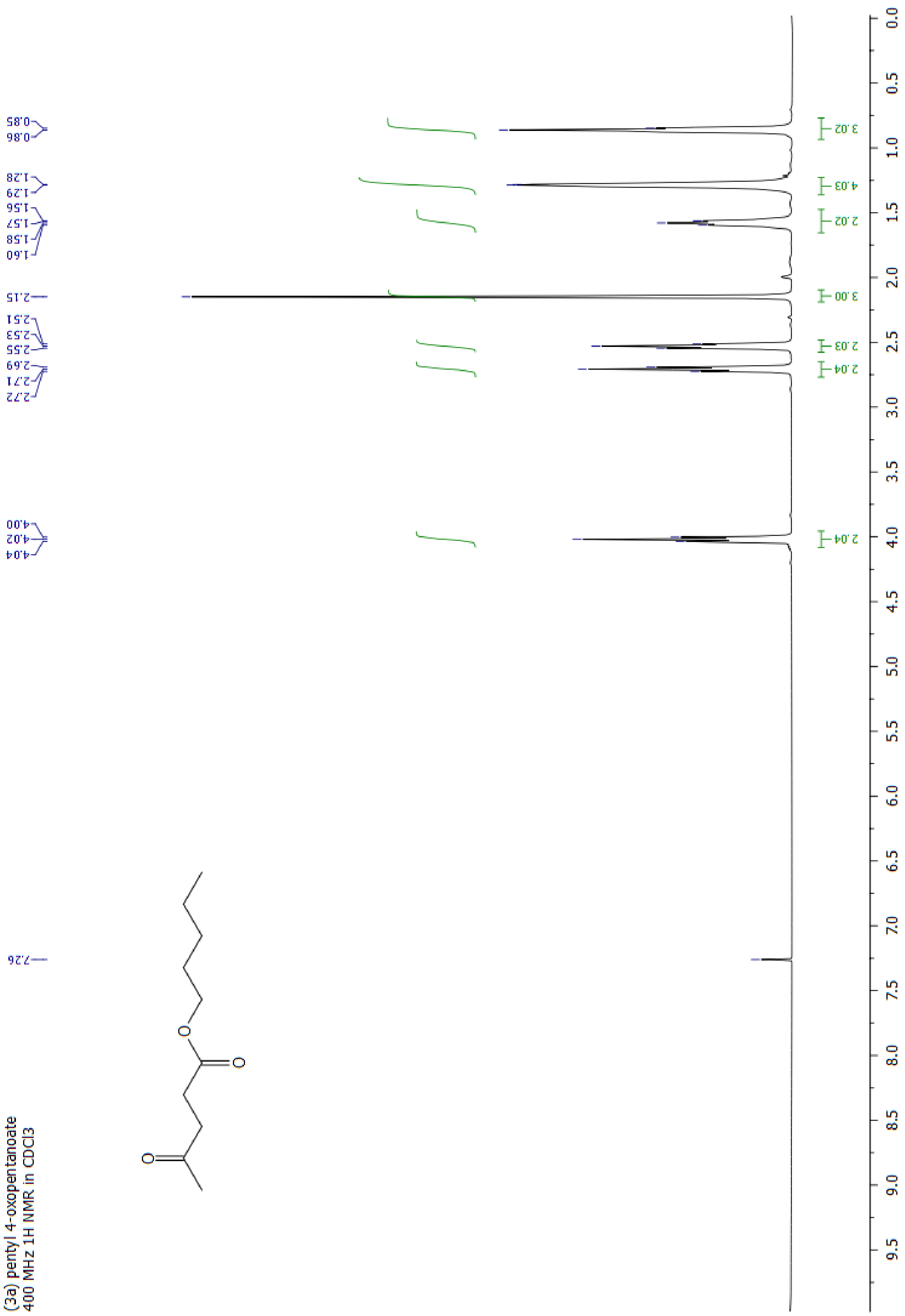

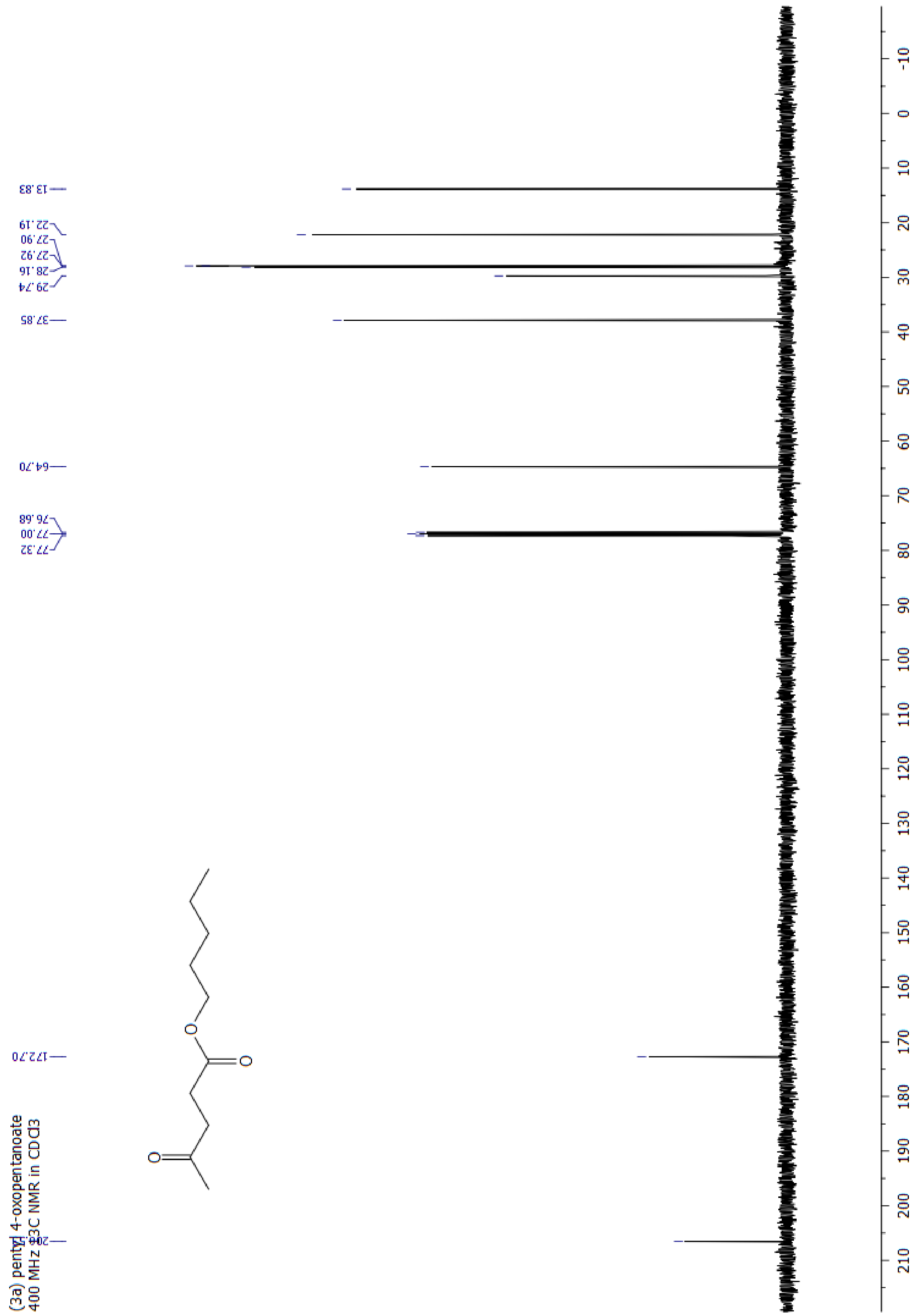
(3a) pentyl 4-oxopentanoate
400 MHz 13C NMR in CDCl3
13.83
22.19
27.90
27.92
28.16
29.74
37.85
64.70
76.68
77.00
77.32
172.70
-10
0
10
20
30
40
50
60
70
80
90
100
110
120
130
140
150
160
170
180
190
200
210

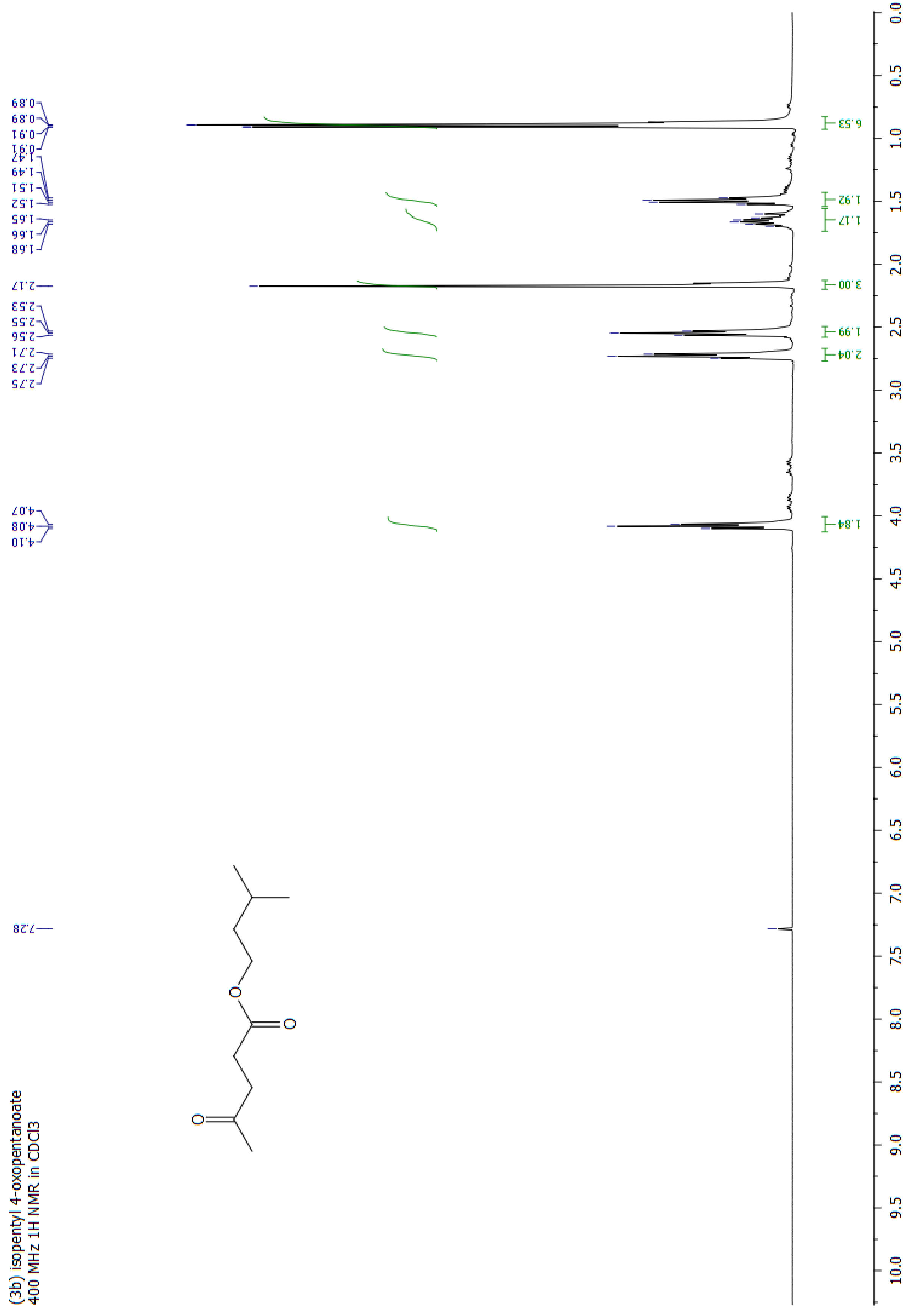
(3b) isopentyl 4-oxopentanoate
400 MHz 1H NMR in CDCl3
0.89
0.89
0.91
0.91
1.47
1.49
1.51
1.52
1.65
1.66
1.68
2.17
2.53
2.55
2.56
2.71
2.73
2.75
4.07
4.08
4.10
7.28
6.53
1.92
1.17
3.00
1.99
2.04
1.84
O
O
O

(3b) isopentyl 4-oxopentanoate
400 MHz 13C NMR in CDCl3

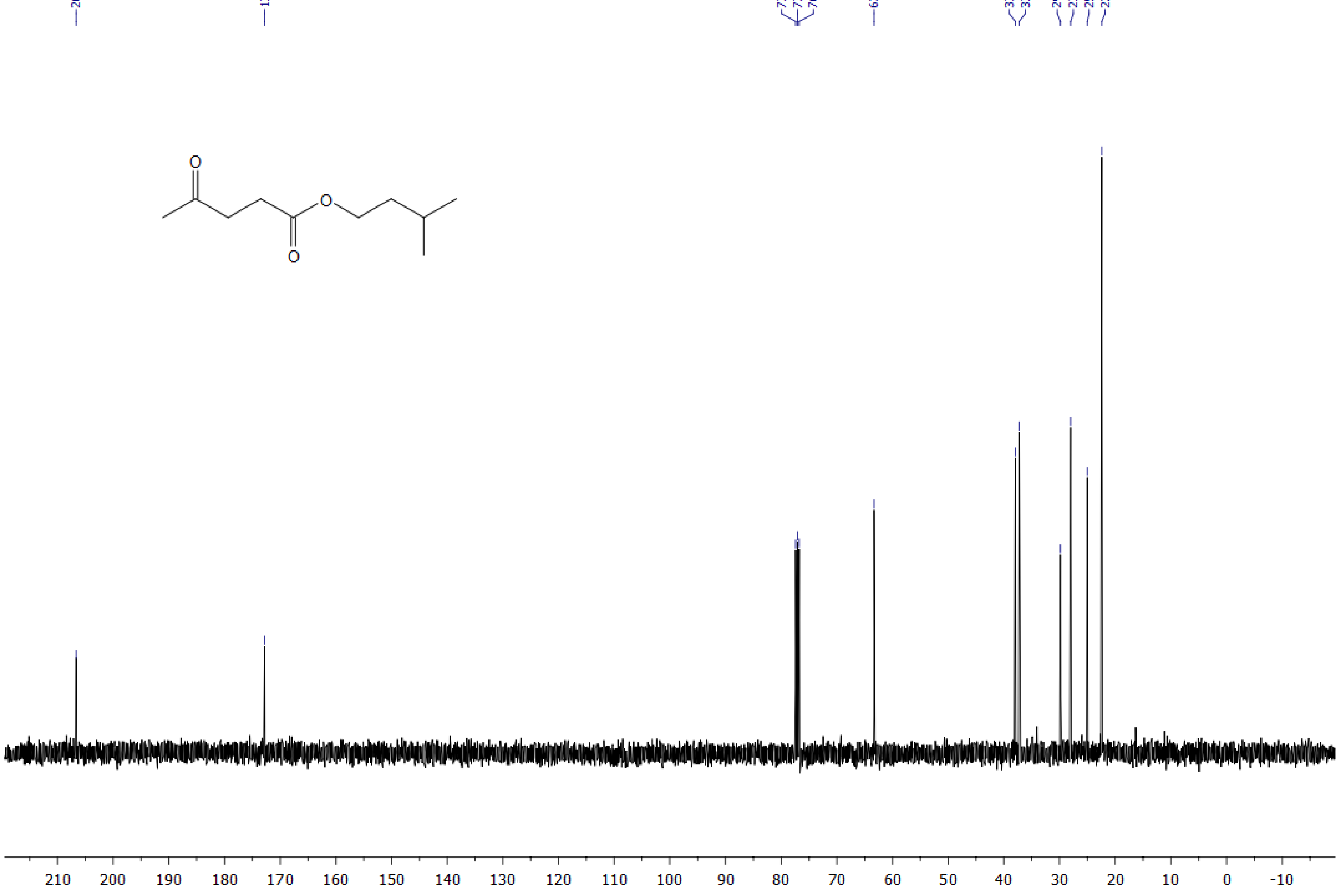

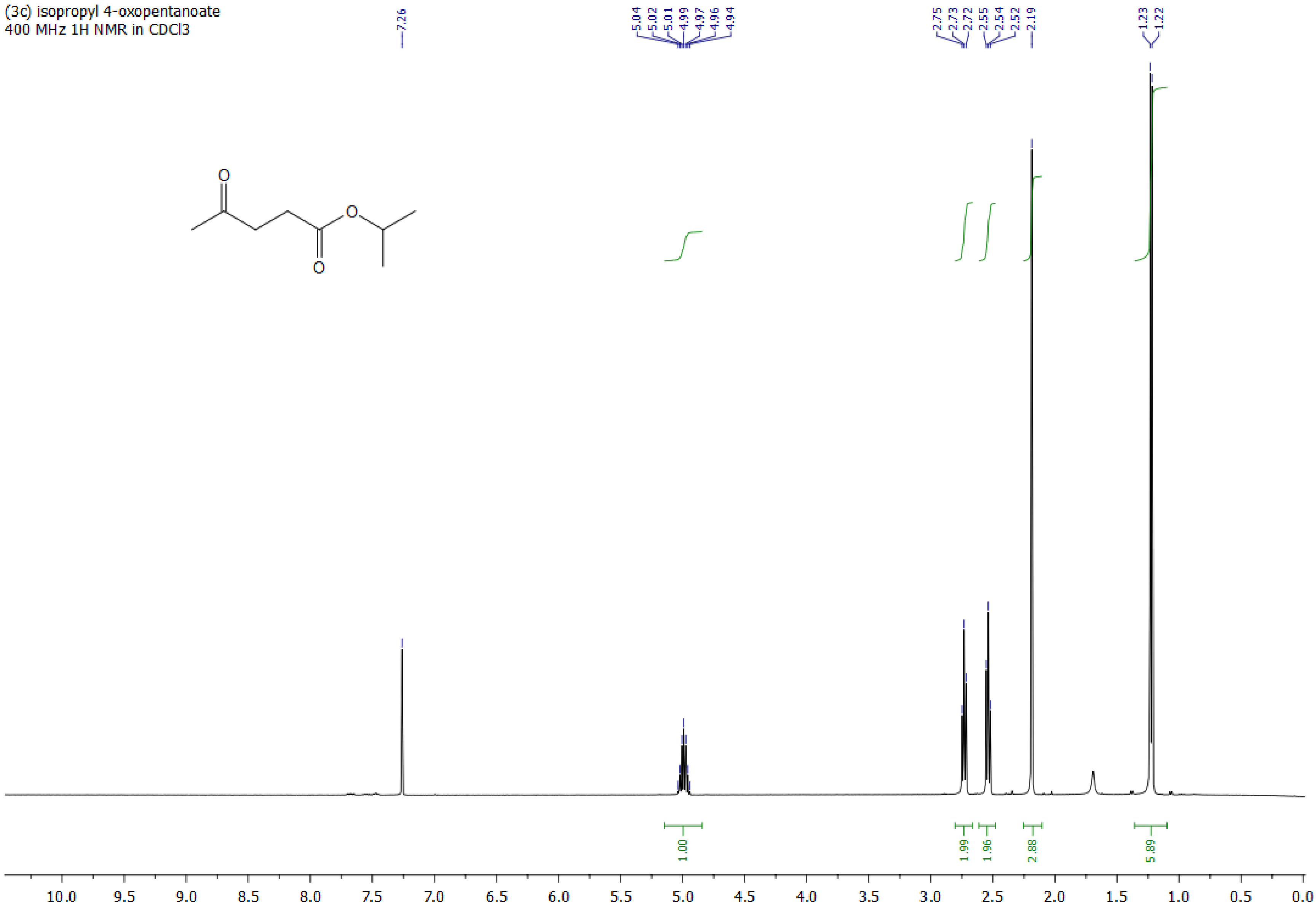

(3c) isopropyl 4-oxopentanoate
400 MHz 1H NMR in CDCl3
7.26
5.04
5.02
5.01
4.99
4.97
4.96
4.94
2.75
2.73
2.72
2.55
2.54
2.52
2.19
1.23
1.22
O
O
O
1.00
1.99
1.96
2.88
5.89
10.0
9.5
9.0
8.5
8.0
7.5
7.0
6.5
6.0
5.5
5.0
4.5
4.0
3.5
3.0
2.5
2.0
1.5
1.0
0.5
0.0

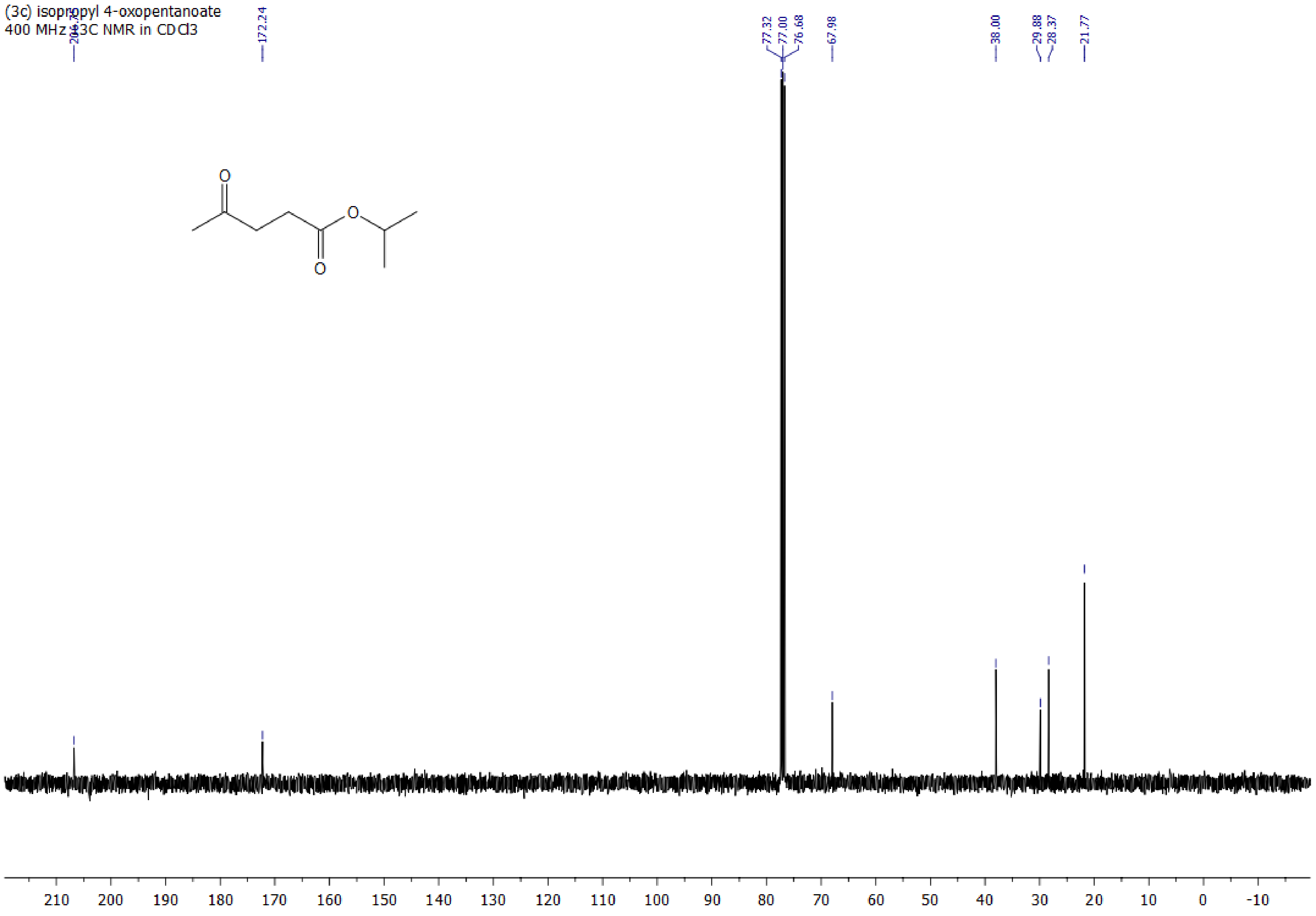

(3c) isopropyl 4-oxopentanoate
400 MHz 13C NMR in CDCl3
172.24
77.32
77.00
76.68
67.98
38.00
29.88
28.37
21.77
O
O
O
210 200 190 180 170 160 150 140 130 120 110 100 90 80 70 60 50 40 30 20 10 0 -10

(3d) (1R,2S,5R)-2-isopropyl-5-methylcyclohexyl 4-oxopentanoate
400 MHz 1H NMR in CDCl3

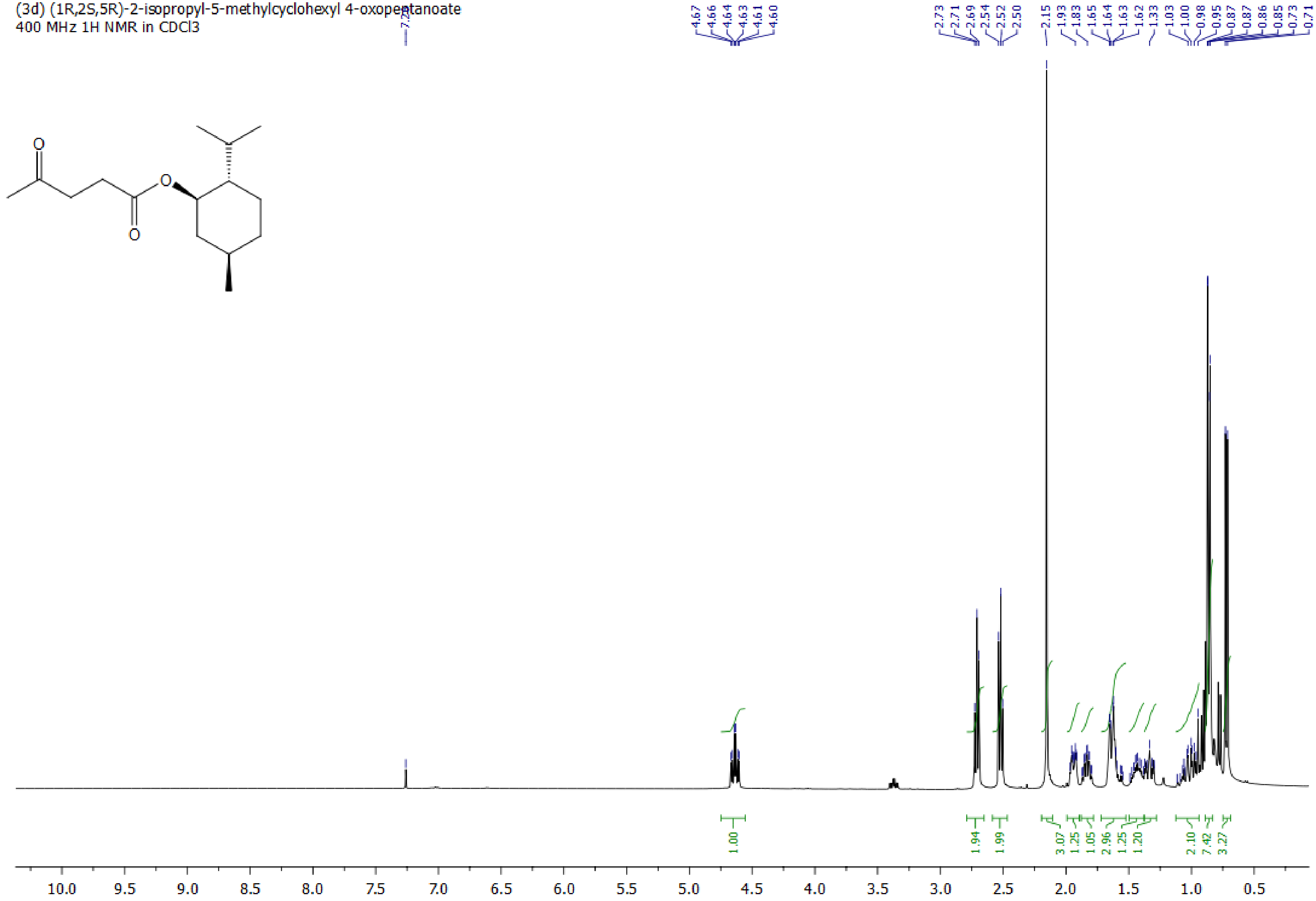

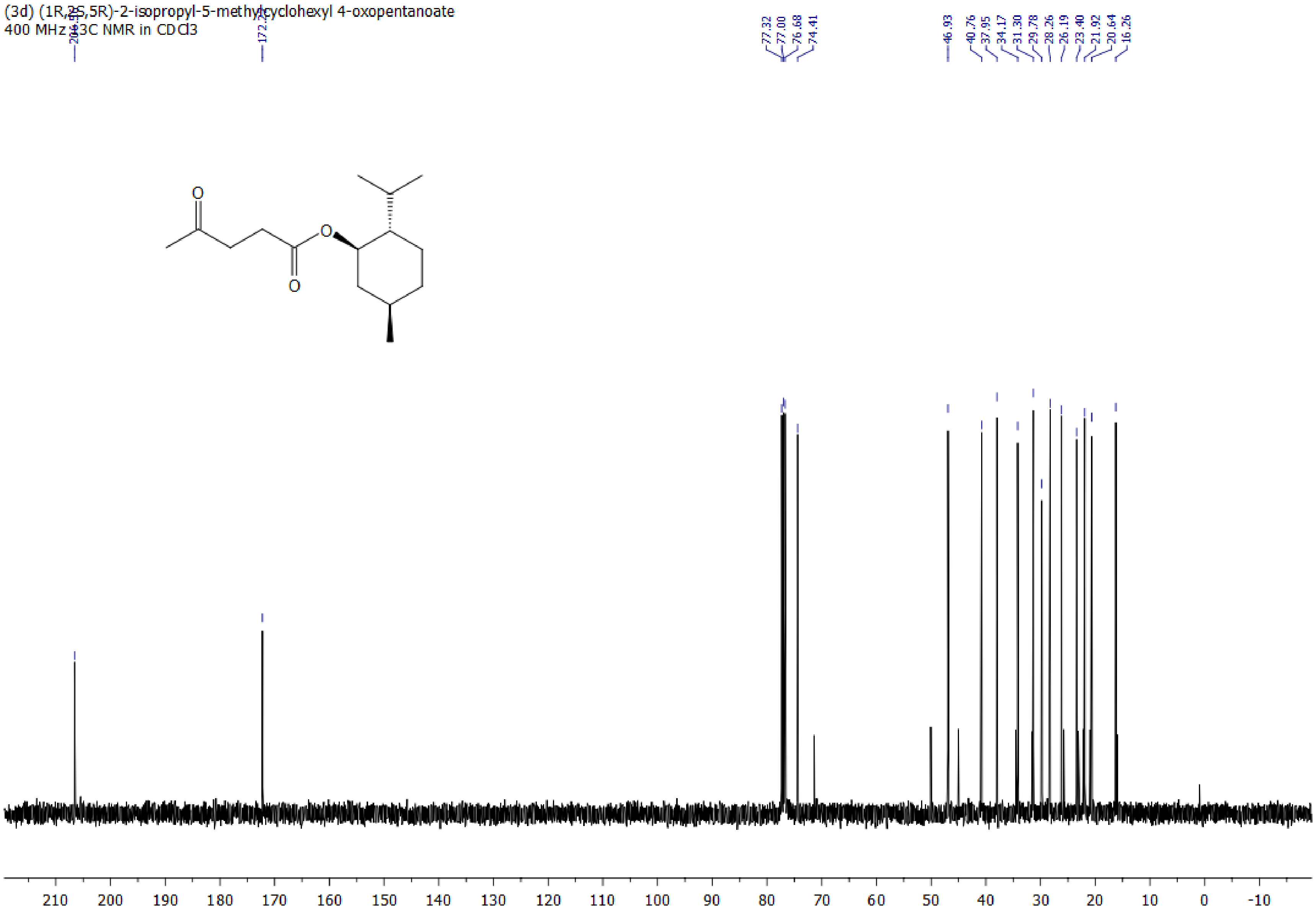

(3d) (1R,2S,5R)-2-isopropyl-5-methylcyclohexyl 4-oxopentanoate
400 MHz 13C NMR in CDCl3
77.32
77.00
76.68
74.41
46.93
40.76
37.95
34.17
31.30
29.78
28.26
26.19
23.40
21.92
20.64
16.26
O
O
O
210
200
190
180
170
160
150
140
130
120
110
100
90
80
70
60
50
40
30
20
10
0
-10
